# Association of P2X_7_ receptor polymorphisms with bone mineral density and osteoporosis risk in a cohort of Dutch fracture patients

**DOI:** 10.1007/s00198-012-2059-x

**Published:** 2012-07-10

**Authors:** A. Wesselius, M. J. L. Bours, Z. Henriksen, S. Syberg, S. Petersen, P. Schwarz, N. R. Jørgensen, S. van Helden, P. C. Dagnelie

**Affiliations:** 1 Department of Epidemiology, School for Public Health and Primary Care (CAPHRI), Maastricht University, Peter Debyeplein 1, P.O. Box 616, 6200 MD Maastricht, The Netherlands; 2 Research Center for Ageing and Osteoporosis, Dept. of Clinical Biochemistry, Glostrup, Copenhagen University Hospital Glostrup, Ndr Ringvej 57-59, 2600 Glostrup, Denmark; 3 Research Center for Ageing and Osteoporosis, Dept. of Medicine, Glostrup, Copenhagen University Hospital Glostrup, Ndr Ringvej 57-59, 2600 Glostrup, Denmark; 4 Department of Trauma Surgery Isala Clinics, Zwolle; formerly Department of Trauma surgery, Maastricht University Medical Centre, PO Box 5800, 6202 AZ Maastricht, The Netherlands

**Keywords:** Bone mineral density, Osteoporosis, P2X7R, Polymorphisms

## Abstract

**Summary:**

The P2X_7_ receptor is thought to be involved in bone physiology in a pro-osteogenic manner. Therefore, we examined associations between genetic variations in the P2X_7_ receptor gene and bone mineral density (BMD). We found an association between four non-synonymous polymorphism of the human P2X_7_ receptor and the risk of osteoporosis.

**Introduction:**

The purpose of this study was to determine whether genetic variation in the P2X_7_ receptor gene (P2RX7) is associated with decreased BMD and risk of osteoporosis in fracture patients.

**Methods:**

Six hundred ninety women and 231 men aged ≥50 years were genotyped for 15 non-synonymous P2RX7 SNPs. BMD was measured at the total hip, lumbar spine and femoral neck.

**Results:**

Four non-synonymous SNPs were associated with BMD. The Ala348Thr gain-of-function polymorphism was associated with increased BMD values at the lumbar spine (*p* = 0.012). Decreased hip BMD values were associated with two loss-of-function SNPs in the P2RX7, i.e., in subjects homozygous for the Glu496Ala polymorphism as well as in subjects carrying at least one variant allele of the Gly150Arg polymorphism (*p* = 0.018 and *p* = 0.011; respectively). In men, we showed that subjects either heterozygous or homozygous for the Gln460Arg gain-of-function polymorphism in the P2RX7 had a significantly 40 % decrease in risk of a lower T-score value (OR = 0.58 [95%CI, 0.33–1.00]).

**Conclusion:**

Thus, genetic aberrations of P2X7R function are associated with lower BMD and increased osteoporosis risk. Therefore, detection of non-synonymous SNPs within the P2RX7 might be useful for osteoporosis risk estimation at an early stage, potentially enabling better osteoporosis prevention and treatment.

**Electronic supplementary material:**

The online version of this article (doi:10.1007/s00198-012-2059-x) contains supplementary material, which is available to authorized users.

## Introduction

Osteoporosis is a skeletal disease characterized by low bone mass and micro-architectural deterioration of bone tissue, leading to bone fragility and increased susceptibility to fracture. One of the most important risk factors of osteoporosis is a positive family history of fracture [1, 2], emphasizing the importance of genetics in osteoporosis.

The purinergic P2X7 receptor (P2X7R) functions as a non-selective ion channel upon activation by high levels (i.e. low millimolar) of extracellular ATP. Sustained stimulation with ATP or repeated stimulation with sequential ATP pulses induces formation of a large pore that permeabilizes the plasma membrane to molecules up to 900 Da.

The P2X7R is demonstrated to be expressed by major bone cell types, including osteoblasts [3–5], osteoclasts [6–8] and osteocytes [9] and the overall effect of a functional P2X7R on bone metabolism is thought to be pro-osteogenic [10, 11]. In vitro studies showed that activation of the P2X7R inhibited bone resorption through initiation of apoptosis of osteoclasts [12]. In osteoblasts, activation of the P2X7R by ATP stimulated the differentiation of osteoblasts and enhanced mineralization [13]. Furthermore, it has been shown that the P2X7R plays an essential role in calcium signalling from osteoblasts to osteoclasts in response to mechanical stimulation [8].

Besides in vitro studies, in vivo studies showed a pro-osteogenic function for the P2X7R on bone metabolism. It was shown that mice lacking the P2X7R had significantly reduced bone mass and increased osteoclast numbers [14]. Furthermore, the P2X7R was shown to be involved in mediation of skeletal mechanotransduction [15].

**Fig. 1:**
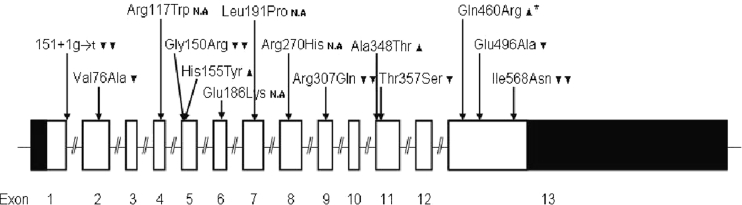
Overview of known functional effects of non-synonymous SNPs in the P2X_7_ recceptor gene. *filled double inverse triangle* Complete loss-of-function polymorphisms, *filled inverse triangle* polymorphisms with reduced receptor function, *filled upright triangle* Polymorphisms with increased receptor function. *N.A.* Not available (no data published on this polymorphism) *filled upright triangle–asterisk* Polymorphism associated with increased receptor function likely caused through linkage with another polymorphism

The P2X7R gene (i.e. P2RX7), located on the long arm of chromosome 12 (12q24), is highly polymorphic, and at least 11 non-synonymous single nucleotide polymorphisms (SNPs) have known effects on P2X7R function, either leading to loss-of-function or gain-of-function (Fig. 1).

Three loss-of-function SNPs (Glu496Ala, Ile568Asn, Arg307Gln) and one gain-of-function SNP (Ala348Thr) were previously shown to be associated with effects on human bone. Both the Glu496Ala and Ile568Asn loss-of-function SNPs showed an association with increased 10-year fracture incidence [16, 17]. The Ile568Asn SNP also showed a positive association with effect of hormone replacement therapy on bone mineral density (BMD) [16]. In addition, the Arg307Gln SNP showed an association with greater cumulative hazard of total hip arthroplasty revision [18], increased rate of bone loss and decreased lumbar spine BMD [19, 20]. Furthermore, subjects harbouring the Ala348Thr SNP were found to have increased BMD values as well as reduced fracture risk [17, 19]. To evaluate a possible predisposition to accelerated bone loss, Jørgensen and co-workers [19] divided subjects into three risk groups (high, intermediate and low) based on a particular combination of several loss-of-function and gain-of-function SNPs with a minor allele frequency between 1 and 3 %. Using this risk model, they demonstrated a highly significant difference between the different risk groups, with individuals belonging to the high-risk group, i.e. individuals with (high risk of) impaired P2X7R function having an increased rate of bone loss.

The above data suggest that the P2RX7 may prove to be an important candidate gene for osteoporosis risk estimation. Therefore, in the present study, we genotyped 15 non-synonymous P2RX7 polymorphisms in a cohort of fracture patients in the southeastern part of the Netherlands, and tested whether genetic variation in this purinergic receptor subtype was associated with BMD, i.e. osteoporosis risk. A fracture cohort was chosen as this is characterized by the high prevalence of osteoporosis [21]. We hypothesized that reduced P2X7R function due to the presence of non-synonymous SNPs in the P2RX7 would be associated with lower BMD values and increased risk of osteoporosis.

## Materials and methods

### Study population and design

The study base for the present study consisted of men and women aged ≥50 years, who visited an osteoporosis outpatient clinic at the Maastricht University Medical Centre (MUMC^+^), the Netherlands, for standard medical care following a recent traumatic or non-traumatic fracture. Fracture patients suffering from a disease of bone metabolism other than osteoporosis (e.g. Paget disease, bone tumours, hyperparathyroidism) were excluded from participation in the present study.

The regular medical follow-up procedure for fracture patients was as follows [21]:

1. Patients who presented with a clinical fracture (confirmed on X-ray) at the emergency unit or who were hospitalized because of a fracture, were invited to the fracture and osteoporosis outpatient clinic;
2. During a first consultation, usually 2–6 weeks following the fracture, besides receiving information about the outpatient clinic and possible treatment regimes, patients were asked to undergo a bone densitometry;
3. During a second consultation, usually 2–4 weeks later, BMD measurement was performed by dual X-ray absorptiometry (DXA) and, in addition, risk factors for falls and osteoporosis were assessed; if indicated, medical treatment for osteoporosis was started according to the Dutch osteoporosis guideline recommendation.

For the present study, we recruited subjects at the outpatient clinic using two different procedures: First, between August 2008 and December 2009, patients at the outpatient clinic received extensive oral and written information about the study during their first visit; then, during a second visit, written informed consent was obtained, and blood samples were collected and stored at −80 °C for subsequent DNA extraction and genotyping. Second, to increase statistical power, saliva was collected from fracture patients who had formerly visited the osteoporosis outpatient clinic before August 2008. Eligible patients for this recruitment procedure were identified using an existing patient database of the osteoporosis outpatient clinic at MUMC^+^, which had been initiated in September 2004. All eligible patients received an information package by mail, which included: (1) a letter to inform patients about the present study; (2) a standard device to collect saliva together with instructions for its use; (3) an informed consent form; and (4) a return envelop with pre-printed address. Patients willing to participate were asked to sign the informed consent form, to donate a small amount of saliva, and to send both of these back to us in the return envelop. Patients, from whom no reaction was received within 2 weeks after the information package had been sent, were contacted once by telephone to increase the response rate.

The study was approved by the ethical committee of the University Hospital Maastricht and Maastricht University, and all participants signed written informed consent after having received proper information about the study before performing any of the study procedures.

### DNA extraction

#### Blood samples

DNA was extracted from blood in an automated procedure using Maxwell 16 DNA purification Kits on the Maxwell 16 instrument (Promega, Madison, WI) 400 μl of blood collected in EDTA-tubes were used and the isolation procedure was performed according to the manufacturer’s instructions.

#### Saliva samples

For collection of a small amount of saliva for DNA extraction, we used a plain cotton swab collection device (Salivette^TM^: Sarstedt AG & Co. Numbrecht, Germany). Upon return, the Salivette^TM^ containing the saliva swab was stored in a refrigerator at 4 °C until DNA extraction. First, the swab kept in the collection tube was centrifuged at 4,000 rpm for 10 min, and the saliva was transferred to a 15 mL Nunc-tube which was kept at 5 °C overnight. Using a pair of sterile tweezers, the swab was then transferred from the collection tube to a 50 mL Nunc-tube; 4 mL sterile water was added and the tube was kept at room temperature overnight. The next day, the swab plus water was transferred back into the collection tube and again centrifuged at 4,000 rpm for 10 min, the saliva yield was again transferred to the 15 mL Nunc-tube already containing the saliva yield from the day before. Next, cells were isolated from the saliva by centrifuging the saliva-containing 15 mL Nunc-tube at 4,000 rpm for 10 min. Subsequently, the supernatant was carefully removed, leaving 600–800 μl over the pellet. DNA extraction was then carried out using Maxwell 16 DNA purification Kits on the Maxwell 16 instrument (Promega, Madison, WI) according to the manufacturer’s instructions.

### Genotyping

The study population was genotyped for 15 non-synonymous SNPs within the P2RX7 that were selected based on their previously published functional effects on the P2X7R, or were found in the dbSNP database for non-synonymous SNPs (Fig. 1). Genotyping was done by Sequenom (Sequenom, Hamburg, Germany) using the Sequenom MassARRAY® iPLEX Gold assay.

To assess the accuracy of the genotyping assay, an internal validation study was performed in which a randomly selected number of samples (*N* = 45) were genotyped a second time, using restriction enzyme digestion of appropriate PCR products or Taqman assay. This was done according to our previously published protocol [22]. When the results were compared with the original genotyping we observed a discrepancy between the two different genotyping methods of ∼4.2 %. The discrepancy appeared to be smaller (∼2.7 %) if the original genotyping with the Sequenom MassARRAY ® iPLEX Gold assay had failed for a maximum of one SNP. Therefore, all subjects in whom the original genotyping had failed for at least two SNPs in the P2RX7 were excluded from statistical analysis.

### Bone density measurements

As part of the standard medical follow-up of fracture patients, bone mineral density (BMD; g/cm^2^) of the lumbar spine (L2–L4), femoral neck, and total hip (trochanter and neck) was assessed by DXA, using the cross-calibrated Hologic QDR 4500 Elite densitometer (Waltham, Massachusetts, USA). BMD T-score values were used to establish the presence or absence of osteoporosis (*T* ≤ −2.5) and osteopenia (*T* < −1 to −2.5). T-score values were calculated using sex specific data from Dutch references.

### Statistical analysis

Deviation of genotype frequencies from those expected under Hardy–Weinberg equilibrium was tested in the non-osteoporotic subjects (i.e. subjects with T-score value greater than −2.5) by the *χ* ^2^ test. Pairwise linkage disequilibrium (LD) between all SNPs was calculated using Haploview v4.0.

Descriptive statistics were used to determine the prevalence of osteoporosis and osteopenia in the cohort of fracture patients, to assess distributions of possible risk factors, including sex, age (in years), body mass index (BMI, in kg/cm^2^), previous fracture (yes/no) and family history of fractures (yes/no), and to describe the occurrence of different fracture types. Other possible risk factors for osteoporosis, such as vitamin D intake, calcium intake, years since menopause and physical activity could not be assessed, since we did not have access to reliable information on these factors.

The software package PLINK was used to test for association between genetic variations and BMD after testing for normal distribution of the data and uniformity of variances using SAS, version 9.1. Preliminary analyses showed that only sex, age and BMI were associated with several SNPs. Therefore all analyses were adjusted for age, sex and BMI. Furthermore, we performed analyses stratified by sex. All analyses include both traumatic and non-traumatic fractures. Both single SNPs and haplotypes were tested for association.

As a confirmatory approach, we used proportional odds logistic regression to estimate the influence of P2RX7 genotypes on the odds of a low BMD T-score value, and thus on osteoporosis risk. For this approach, quintiles of the population were defined based on BMD T-score values. The proportional odds assumption was tested using the chi-square score test. Again, analyses were performed for the total population as well as stratified by sex. This was done by the use of SAS, version 9.1.

For all analyses, *p* values lower than 0.05 were considered statistically significant.

## Results

### Study population

**Fig. 2:**
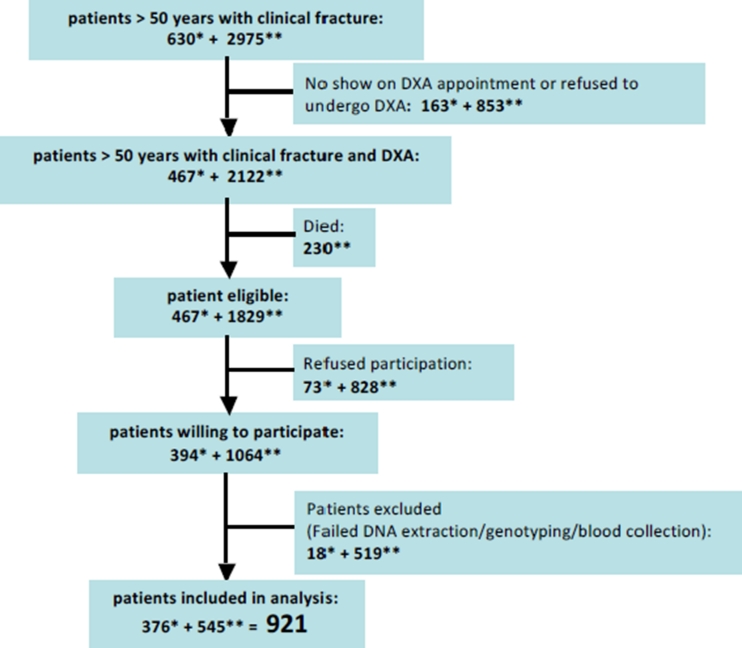
Flowchart of patient recruitment for the present study. *Number of patients recruited during phase 1, i.e. between August 2008 and December 2009. **Number of patients recruited during phase 2, i.e. between January and July 2010

Of the 630 patients with a recent fracture who were invited to the osteoporosis outpatient clinic between August 2008 and December 2009, 467 (74.1 %) were willing to undergo bone densitometry. Of these, during their second consultation at the osteoporosis outpatient clinic, 394 (84.4 %) were willing to donate blood. The collection of blood failed for 13 (3.3 %) patients and genotyping for 5 (1.3 %) patients (Fig. 2).

Of the 2,975 fracture patients who had formerly visited the osteoporosis outpatient clinic between September 2004 and August 2008, 2,122 (71.3 %) had undergone bone densitometry. Two hundred thirty (10.8 %) of these patients had died in the meantime. Of the remaining 1,892 former fracture patients who were invited by mail to participate in the present study, 1,064 (58.2 %) gave informed consent and returned saliva samples. DNA extraction failed for 27 (2.5 %) samples (Fig. 2). Based on our internal validation study (see “Materials and Methods”), genotyping failure was defined as having ≥2 missing SNPs out of a total of 15 SNPs in the P2RX7; based on this, genotyping failed for 492 (46.2 %) samples (Fig. 2). In total, 921 samples were successfully genotyped and used for subsequent analyses.

Characteristics of the 921 participants are listed in Table 1. The final study population consisted of 690 women aged 65.5 ± 9.8 years (mean ± SD) and 231 men aged 63.5 ± 9.6 years. The prevalence of osteoporosis was 32.2 % among women and 26.4 % among men, and the prevalence of osteopenia was 48.0 % among women and 42.0 % among men. Hip fractures and fractures of the humerus were most common among subjects suffering from osteoporosis (12.2 % and 15.7 %; respectively), whereas other common osteoporotic fractures, i.e., fractures of the lumbar spine and wrist, were most frequent in subjects suffering from osteopenia (4.8 and 30.0 %; respectively). Fracture of the ankle was the most common fracture among the non-osteoporotic fractures (Supplemental table 1) No differences in baseline characteristics were observed between the two different types of data collected (i.e. blood and saliva). Furthermore, no differences in baseline characteristics were observed between subjects included in the analyses and subjects excluded based on the internal validation study.

**Table 1:** Characteristics of the study population

Characteristics	Total (*N* = 921) mean (SD)	Men (*N* = 231) mean (SD)	Women (*N* = 690) mean (SD)
Age (Y)	65.0 (9.8)	63.5 (9.6)	65.5 (9.8)
Weight (kg)	72.5 (13.8)	82.29 (12.4)	69.2 (12.6)
Height (cm)	165.8 (9.1)	175.7 (7.3)	162.5 (6.9)
BMI (kg/m^2^)	26.3 (4.2)	26.6 (3.7)	26.2 (4.4)
Femoral neck BMD (g/cm^2^)	0.69 (0.13)	0.76 (0.13)	0.66 (0.12)
Total hip BMD (g/cm^2^)	0.84 (0.15)	0.95 (0.15)	0.80 (0.13)
Lumbar spine BMD (g/cm^2^)	0.93 (0.17)	0.98 (0.17)	0.91 (0.17)
Osteoporosis (% (*N*))	30.7 (283)	26.4 (61)	32.2 (222)
Osteopenia (% (*N*))	46.5 (428)	42.0 (97)	48.0 (331)
Normal BMD (% (*N*))	22.8 (210)	31.6 (73)	19.8 (137)
Type of fracture	Osteoporosis (% (*N*))	Osteopenia (% (*N*))	Normal BMD (% (*N*))
Humerus (*N* = 108)	15.7 (40)	11.6 (46)	11.2 (22)
Femur (*N* = 75)	12.2 (31)	8.8 (35)	4.6 (9)
Lumbar spine (*N* = 38)	4.3 (11)	4.8 (19)	4.1 (8)
Wrist (*N* = 208)	25.2 (64)	30.0 (119)	12.7 (25)
Other fracture (*N* = 419)	42.5 (108)	45.6 (178)	67.5 (133)

^a^ *BMI* body mass index

^b^ *BMD* bone mineral density

^c^Osteoporosis defined by BMD T-score values, *T* ≤ −2.5

^d^Osteopenia defined by BMD T-score values, *T* < −1 to −2.5.

### P2X_7_ genotypes

Minor allele frequency and information on HWE of the 15 genotyped non-synonymous SNPs within the P2RX7 in non-osteoporotic subjects are shown in Table 2. SNPs were found to be in HWE except for the Ala348Thr and Val76Ala polymorphisms.

**Table 2:** P2RX7 SNPs and HWE in subjects with a normal T-score, i.e. T-score > −2.5

rs number	Base change	Polymorphism	MAF	HWE *p* value
rs35933842	151 + 1 g→t	Null Allele	0.009	1
rs17525809	253T→C	Val76Ala	0.046	0.010
rs28360445	375C→T	Arg117Trp	0	1
rs28360447	474G→A	Gly150Arg	0.016	0.115
rs208294	489C→T	His155Tyr	0.447	0.335
rs28360451	582G>A	Glu186Lys	0	1
rs28360452	598T>C	Leu191Pro	0	1
n.a.	699C→T	Null Allele	0.039	0.617
rs16950860	834T→C	Arg270Cys	0	1
rs28360457	946G→A	Arg307Gln	0.006	1
rs1718119	1068G→A	Ala348Thr	0.381	<0.001
rs2230911	1096C→G	Thr357Ser	0.061	0.282
rs2230912	1405A→G	Gln460Arg	0.169	0.065
rs3751143	1513A→C	Glu496Ala	0.179	0.892
rs1653624	1729T→A	Ile568Asn	0.033	1

^a^ *MAF* minor allele frequency

^b^ *HWE* Hardy–Weinberg equilibrium

^c^ *N/A* not available

### Association of P2RX7 genotypes with bone mineral density

Table 3 shows the association of different P2RX7 genotypes with bone mineral density. BMD values at the lumbar spine were significantly higher in subjects homozygous for the variant alleles (i.e. TT genotype) of the Ala348Thr gain-of-function polymorphism than in subjects having the other two genotypes (recessive model: *p* = 0.016). The proportional odds logistic regression showed that the odds of a lower T-score (i.e. the risk of osteoporosis) at the lumbar spine was decreased by approximately 25 % in subjects at least one wild-type allele of the Ala348Thr polymorphism compared to subjects homozygous for the variant allele (lumbar spine OR = 0.75 [95%CI, 0.55–0.86]). Sex stratified analyses showed that in women BMD values at both the lumbar spine was significantly higher among women homozygous for the variant allele (recessive model; *p* = 0.0025). In men, no significant differences in BMD at the hip or spine were found between Ala348Thr genotypes.

**Table 3:** BMD values for the individual genotypes for each single SNP and the risk model

Ala348Thr	CC	CT	TT	*p* value^a^ additive	*p* value^b^ recessive	*p* value^c^ dominant
*N*	363	364	153			
BMD TH (g/cm^2^)	0.84 (0.15)	0.83 (0.15)	0.85 (0.15)	0.7435	0.7332	0.8256
BMD LS (g/cm^2^)	0.93 (0.16)	0.91 (0.16)	0.95 (0.18)	0.5836	0.0160	0.2948
BMD FN (g/cm^2^)	0.69 (0.13)	0.67 (0.12)	0.70 (0.12)	0.2633	0.7553	0.1577
Female						
*N*	265	268	119			
BMD TH (g/cm^2^)	0.80 (0.14)	0.79 (0.13)	0.81 (0.14)	0.2896	0.0724	0.2719
BMD LS (g/cm^2^)	0.91 (0.15)	0.89 (0.16)	0.94 (0.18)	0.1490	0.0025	0.8262
BMD FN (g/cm^2^)	0.67 (0.12)	0.65 (0.11)	0.68 (0.12)	0.1461	0.7578	0.0544
Male						
*N*	94	92	34			
BMD TH (g/cm^2^)	0.94 (0.15)	0.94 (0.15)	0.98 (0.14)	0.3570	0.7431	0.2773
BMD LS (g/cm^2^)	1.00 (0.18)	0.97 (0.16)	0.97 (0.17)	0.2036	0.7895	0.1018
BMD FN (g/cm^2^)	0.75 (0.13)	0.75 (0.13)	0.77 (0.10)	0.8439	0.9908	0.7834
Glu496Ala	TT	GT	GG			
*N*	619	264	34			
BMD TH (g/cm^2^)	0.84 (0.16)	0.83 (0.14)	0.79 (0.16)	0.6841	0.1887	0.9674
BMD LS (g/cm^2^)	0.93 (0.17)	0.92 (0.16)	0.89 (0.13)	0.0662	0.0180	0.2228
BMD FN (g/cm^2^)	0.69 (0.13)	0.68 (0.12)	0.66 (0.13)	0.9628	0.7956	0.9621
Female						
*N*	455	200	24			
BMD TH (g/cm^2^)	0.80 (0.14)	0.80 (0.13)	0.74 (0.11)	0.9388	0.0376	0.459
BMD LS (g/cm^2^)	0.91 (0.17)	0.90 (0.15)	0.87 (0.13)	0.1211	0.0172	0.3846
BMD FN (g/cm^2^)	0.66 (0.12)	0.67 (0.12)	0.63 (0.10)	0.7330	0.4162	0.4677
Male						
*N*	159	63	7			
BMD TH (g/cm^2^)	0.95 (0.16)	0.93 (0.14)	1.00 (0.14)	0.5303	0.4933	0.3242
BMD LS (g/cm^2^)	0.98 (0.17)	0.97 (0.16)	0.95 (0.15)	0.2566	0.7161	0.2378
BMD FN (g/cm^2^)	0.76 (0.13)	0.74 (0.12)	0.80 (0.13)	0.5421	0.4232	0.3132
Gly150Arg	GG	AG	AA			
*N*	885	31	2			
BMD TH (g/cm^2^)	0.84 (0.15)	0.81 (0.17)	0.64 (0.35)	0.8351	0.633	0.7295
BMD LS (g/cm^2^)	0.93 (0.17)	0.87 (0.17)	0.78 (0.32)	0.0109	0.6247	0.0081
BMD FN (g/cm^2^)	0.69 (0.12)	0.66 (0.16)	0.56 (0.24)	0.8723	0.8227	0.9056
Female						
*N*	655	24	2			
BMD TH (g/cm^2^)	0.80 (0.13)	0.77 (0.15)	0.64 (0.35)	0.9372	0.9523	0.6024
BMD LS (g/cm^2^)	0.91 (0.16)	0.84 (0.16)	0.79 (0.32)	0.0377	0.6332	0.0299
BMD FN (g/cm^2^)	0.67 (0.11)	0.65 (0.16)	0.56 (0.24)	0.5539	0.8128	0.4693
Male						
*N*	223	7				
BMD TH (g/cm^2^)	0.95 (0.15)	0.94 (0.21)		0.6119		
BMD LS (g/cm^2^)	0.98 (0.17)	1.01 (0.18)		0.1062		
BMD FN (g/cm^2^)	0.76 (0.13)	0.71 (0.15)		0.1896		
His155Tyr	GG	AG	AA			
*N*	294	429	189			
BMD TH (g/cm^2^)	0.84 (0.15)	0.83 (0.15)	0.83 (0.16)	0.1452	0.6716	0.0609
BMD LS (g/cm^2^)	0.92 (0.16)	0.93 (0.16)	0.93 (0.18)	0.6359	0.8678	0.3827
BMD FN (g/cm^2^)	0.69 (0.13)	0.69 (0.12)	0.68 (0.13)	0.0268	0.6602	0.0024
Female						
*N*	215	313	148			
BMD TH (g/cm^2^)	0.80 (0.13)	0.80 (0.13)	0.80 (0.14)	0.1670	0.3274	0.1977
BMD LS (g/cm^2^)	0.90 (0.16)	0.91 (0.15)	0.91 (0.18)	0.4770	0.8503	0.2009
BMD FN (g/cm^2^)	0.67 (0.12)	0.67 (0.11)	0.66 (0.11)	0.0903	0.3888	0.0601
Male						
*N*	75	115	38			
BMD TH (g/cm^2^)	0.95 (0.15)	0.94 (0.15)	0.95 (0.15)	0.5513	0.5115	0.1627
BMD LS (g/cm^2^)	0.98 (0.17)	0.98 (0.17)	0.98 (0.17)	0.7666	0.9679	0.6419
BMD FN (g/cm^2^)	0.77 (0.14)	0.74 (0.12)	0.77 (0.14)	0.1398	0.6249	0.5286
Gln460Arg	AA	AG	GG			
*N*	653	229	36			
BMD TH (g/cm^2^)	0.83 (0.15)	0.84 (0.16)	0.86 (0.16)	0.6586	0.7918	0.1577
BMD LS (g/cm^2^)	0.92 (0.17)	0.94 (0.18)	0.90 (0.17)	0.5371	0.6092	0.2910
BMD FN (g/cm^2^)	0.69 (0.12)	0.69 (0.13)	0.70 (0.13)	0.3625	0.6986	0.2071
Female	AA	AG	GG			
*N*	479	177	32			
BMD TH (g/cm^2^)	0.80 (0.13)	0.79 (0.14)	0.84 (0.15)	0.1347	0.9245	0.0724
BMD LS (g/cm^2^)	0.91 (0.16)	0.92 (0.18)	0.90 (0.18)	0.4535	0.7098	0.2751
BMD FN (g/cm^2^)	0.67 (0.12)	0.66 (0.12)	0.68 (0.11)	0.0711	0.9123	0.4677
Male	AA	AG	GG			
*N*	173	52	4			
BMD TH (g/cm^2^)	0.93 (0.16)	0.97 (0.14)	1.07 (0.01)	0.1314	0.3921	0.1577
BMD LS (g/cm^2^)	0.98 (0.17)	0.99 (0.17)	0.89 (0.04)	0.9809	0.2662	0.7563
BMD FN (g/cm^2^)	0.75 (0.13)	0.78 (0.12)	0.88 (0.02)	0.2407	0.2237	0.3515

*p* values are shown for PLINK association analysis for the bone mineral density (BMD) parameters adjusted for age, BMI and sex. LS: lumbar spine; FN: femoral neck; TH: total hip

^a^Numbers are means (SD)

^b^All analyses are adjusted for age, BMI and sex

The Glu496Ala loss-of-function polymorphism was associated with a decreased lumbar spine BMD: subjects homozygous for the variant alleles of the Glu496Ala polymorphism (i.e. GG genotype) showed a lower BMD value compared to both heterozygous and wild-type subjects (recessive model, *p* = 0.018). In women, besides lumbar spine values, total hip BMD values were significantly reduced in subjects homozygous for the variant allele of the Glu496Ala loss-of-function polymorphism (recessive model, *p* = 0.017 and 0.038, respectively). The proportional odds logistic regression confirmed the findings in women for the total hip, showing an increased odds of lower BMD in women homozygous for the variant allele compared to women carrying at least one wild-type allele (OR = 2.47 [95%CI, 1.15–5.32]). No significant differences between genotypes of the Glu496Ala polymorphism were observed in men.

Subjects carrying the variant allele of the Gly150Arg polymorphism showed reduced BMD values at all sites. This reduction was significant at the lumbar spine (additive model, *p* = 0.011), and the proportional odds logistic regression confirmed a 1.78 times elevated odds of lower T-score values and thus an increased risk of osteoporosis (OR = 1.28, 95% CI = 1.03–3.40). Similar results were found in the stratified analyses for women (additive model, *p* = 0.0377; odds model, OR = 2.28 [95% CI = 1.10–4.72]).

Significantly reduced femoral neck BMD values were observed for subject carrying the variant allele of the His155Tyr polymorphism (additive model, 0.027). This result was not statistically significant in the analyses stratified by gender.

Although overall analyses showed no statistically significant effect of the Gln460Arg polymorphism, analyses stratified by sex showed a 40 % decreased odds of a lower T-score at the femoral neck (OR = 0.58 [95%CI, 0.33–1.00]) in men carrying at least one variant allele of the Gln460Arg polymorphism (i.e. AG and GG genotypes) compared to wild-type men.

None of the other polymorphisms showed an association with BMD at any site (data not shown).

### Linkage disequilibrium between SNPs

**Fig. 3:**
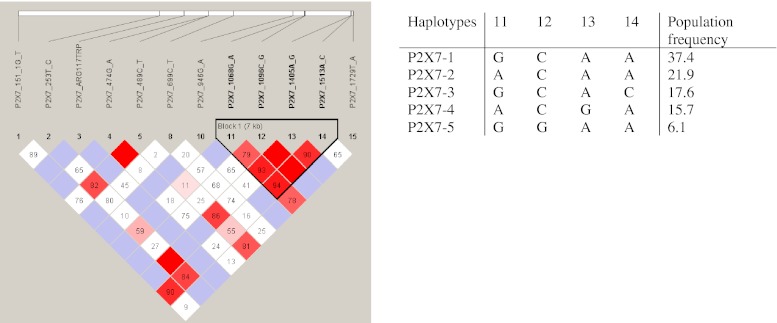
Linkage disequilibrium between P2X7 SNPs. Plot of relative *D*′/LOD scores between P2X7 SNPs from European Caucasian subjects produced by the haploview program. Standard color scheme is displayed: *bright red* (*D*′ = 1; LOD ≥ 2), *blue* (*D*′ = 1; LOD < 2), *shade of pink/red* (*D*′ < 1; LOD ≤ 2), *white* (*D*′ < 1; LOD < 2)

Four polymorphisms Ala348Thr, Thr357Ser, Gln460Arg and Glu496Ala showed strong LD (Fig. 2) and therefore haplotypes could be reconstructed. The constructed haplotype contained only five variants covering 99 % of the genotyped subjects, which have been termed P2X7-1 to P2X7-5 (Fig. 3).

The most frequent haplotype was the P2X7-1 variant, accounting for 37.4 % of the alleles. This haplotype was defined as wild-type. The P2X7-2 and P2X7-4 variants contained the variant allele of the Ala348Thr polymorphism and accounted for 24.9 and 15.7 % of the alleles, respectively. Besides the Ala348Thr polymorphism, the P2X7-4 variant also contained the variant allele of the Gln460Arg polymorphism. The P2X7-3 and P2X7-5 variants contained the loss-of-function polymorphisms Thr357Ser and Glu496Ala, respectively. Strong linkage disequilibrium was found between the Glu496Ala polymorphism and the null allele (*D*′ = 0.90; Fig. 3). Furthermore, linkage disequilibrium was observed between the Gln460Arg polymorphism and the His155Tyr gain-of-function polymorphism (*D*′ = 0.86).

### Association of P2RX7 haplotypes with bone mineral density

Haplotype analysis of the association between BMD and haplotypes showed decreased BMD values in subjects with haplotype P2X7-3. Assuming an additive model this decrease was significant at the lumbar spine (*p* = 0.035). The proportional odds model showed a significantly increased odds of a lower T-score (OR = 2.09 [95%CI, 1.06–4.11]) for subjects with haplotype P2X7-3 compared to wild-type subjects (i.e. subjects having haplotype P2X7-1). Gender-stratified analyses showed no association of any of the haplotypes with BMD.

## Discussion

Within a cohort of Dutch fracture patients we investigated 15 non-synonymous SNPs within the P2RX7 in association with osteoporosis. Results showed that the Ala348Thr gain-of-function polymorphism in the P2RX7 was associated with increased lumbar spine BMD values. We also observed significant associations between BMD values and two loss-of-function SNPs in the P2RX7, that is, decreased hip BMD values were found in subject homozygous for the Glu496Ala polymorphism as well as subjects carrying at least one variant allele of the Gly150Arg polymorphism. In men we found that subjects either heterozygous or homozygous for the Gln460Arg gain-of-function polymorphism in the P2RX7 had a significantly decreased risk of osteoporosis.

The Glu186Lys, Leu191Pro and the Arg270Cys polymorphisms were not present in the studied population. The allele frequencies for the remaining 12 SNPs in our population were almost identical to previously published data [17, 19]. In non-osteoporotic subjects, SNPs were shown to be in HWE, except the Ala348Thr and Val76Ala polymorphisms which showed significant deviation from HWE. Since the internal validation study, in which we repeated the genotyping in a random sub-sample of our study population, indicated adequate accuracy for subjects with <2 missing SNPs in the P2RX7, genotyping errors are a very unlikely explanation for the observed deviation from HWE. Since our recruitment strategy was based on the presence of a fracture, the deviation from HWE for the Ala348Thr polymorphism can most likely be explained by the recently observed association of this polymorphism with fracture incidence [19].

The Ala348Thr, His155Tyr and Gln460Arg have all been demonstrated to be gain-of-function polymorphisms of the P2X7R [23–26]. Cells containing the variant allele of the Ala348Thr polymorphism showed increased pore formation and channel function of the P2X7R [25, 26]. In line with these in vitro studies, and consistent with previously reported data from two Danish cohort studies [17, 19], we found that the 348Thr allele was associated with increased lumbar spine BMD values. In contrast, the variant allele of the His155Tyr polymorphism was found to be associated with decreased femoral neck BMD values. This result is in contrast with previous findings in both in vitro and human association studies [17, 23, 25]. Therefore, further research will be needed to elucidate the association between the His155Tyr polymorphism and BMD values.

The third gain-of function polymorphism, the variant allele of the Gln460Arg polymorphism, showed a significant association with osteoporosis in men. Similar results were reported by the groups of Langdahl et al. [17]. Although in vitro studies showed that the Gln460Arg polymorphism had no major functional effect on the P2X7R [23–25], it has been suggested as an indicator of the most pronounced increase in P2X7R function, as it has been shown to be coinherited with three other gain-of-function polymorphisms (Ala348Thr, His155Tyr and His270Arg) [24]. However, haplotype analysis in the present study showed that haplotype P2X7-4, containing both the Ala348Thr and Gln460Arg polymorphisms did not show increased BMD values, suggesting that the gain-of-function effect of Gln460Arg polymorphism is not the consequence of Ala348Thr polymorphism. Furthermore, we did not detect a gain-of-function effect of the His155Tyr polymorphism in our Dutch fracture cohort. Since research showed that the P2X4R is co-expressed with and closely situated to the P2X7R, it can be speculated that the observed gain-of-function effect of the Gln460Arg polymorphisms is actually the consequence of polymorphisms within the P2XR4.

In in vitro studies, complete loss of P2X7R function has been shown for the Arg307Gln, Ile568Asn, Gly150Arg polymorphisms [27–31]. Of these, the Arg307Gln was previously reported to be significantly associated with decreased lumbar spine BMD values and greater bone loss in the hip, in postmenopausal women [19, 20]. In line with these previous reports, we observed non-significant decreased BMD values at all sites in subjects carrying the variant allele of the Arg307Gln polymorphism relative to wild-type subjects (data not shown). The low number of subjects detected carrying the 307Gln allele (*N* = 11) may have prohibited formal statistical evaluation of the association between the Arg307Gln polymorphism and decreased BMD values.

In Danish postmenopausal women, the Ile568Asn loss-of-function polymorphism was associated with 10-year vertebral fracture incidence and increased rate of bone loss [16]. In contrast, we, like two other association studies [17, 20], did not find any association between the Ile568Asn loss-of-function polymorphism and BMD. However, only two women homozygous for the variant allele could be identified in this study.

Since both the Arg307Gln and Ile568Asn were previously showed to be associated with either decreased BMD and/or fracture risk, the observed low prevalence of these SNPs in our fracture cohort is contrary to our expectations.

The variant allele of the Gly150Arg polymorphism in our study was associated with decreased lumbar spine BMD, supporting the results found by Husted and colleagues [17], who observed reduced total hip BMD values in subjects carrying the 150Arg allele. This effect on BMD might be explained by its complete loss-of-function effect on the P2X7R [25, 32].

In line with several in vitro studies which showed that the variant allele of the Glu496Ala polymorphism was associated with a loss of receptor function [16, 23, 28, 33, 34], human cohort studies showed this polymorphism to be associated with decreased BMD values in both men and women [17] and increased fracture incidence over 10 years after menopause [16]. In concordance with these findings, we also found significantly decreased BMD values at the total hip in women with at least one variant allele of the Glu496Ala polymorphism. Furthermore, analysis of haplotypes containing the Glu496Ala polymorphism (i.e. haplotype P2X7-3) also showed a significant association with decreased BMD values at the lumbar spine. This is in line with the results found by Stokes et al. [24], indicating that this haplotype is associated with decreased receptor function.

The studied P2X7R SNPs mostly affect the lumbar spine. Since bone turnover is primarily taking place on the bone surfaces and the changes in BMD due to the P2X7R SNPs are relatively small, one possible explanation for affecting this particular skeletal site could be that trabecular bone is lost more rapidly than cortical bone. As the amount of trabecular bone is higher in the vertebrae than in the hip, the bone loss will be most pronounced in the vertebral spine.

The present study has several limitations. First, our study population is not population-based, as the recruitment strategy was based on the presence of a fracture. The prevalence of low BMD is, therefore, expected to be higher in our study population than in the general population. Furthermore, if the studied P2 receptor SNPs could affect fracture risk either directly or indirectly (independent of BMD) then the prevalence of this particular SNP would also be expected to be higher in our study sample than in the general population. This could potentially lead to bias in the results in the sense that extrapolation to the general population is compromised. Whether this bias may have lead to an over- or underestimation of the true association in the general population remains unclear, since the magnitude and direction of the association of both the SNP and BMD with fracture is unknown. However, for the above reason care should be taken when translating our findings to the general population.

Second, although statistical power was adequate to detect effects of common polymorphisms in the P2RX7, it was limited for detecting small effect sizes for the more rare polymorphisms, especially in men. Third, we did not have access to reliable information on additional risk factors for osteoporosis, such as vitamin D intake, calcium intake, years since menopause and physical activity. These factors could therefore not be taken into account in our analyses. Especially a possible interaction between physical activity and P2X_7_ SNPs in relation to osteoporosis risk would have been interesting to investigate, since previous animal studies using P2X_7_ knock-out models demonstrated that bone formation in response to mechanical loading as a result of enhanced production of prostaglandin-E_2_ via P2X7R activation was diminished in P2X_7_ knock-outs [15, 35].

Finally, in view of multiple statistical testing it could be debated whether, for instance, Bonferroni *p* value adjustments should have been applied. However, it previously has been argued that the use of Bonferroni *p* value adjustments is impractical and likely too conservative when testing a priori hypotheses [36]. Since we were able to formulate plausible a priori hypotheses regarding most of the P2RX7 SNPs, based on data from previous studies, we did not apply Bonferroni correction in our analyses. Furthermore, almost all associations observed in our study were in accordance with previously published functional effects of the polymorphisms, further strengthening the plausibility of our results. If, however, we had adjusted for the number of independent polymorphisms (*n* = 12) the significance level would have been 0.0042. In that case, most of the observed associations between the individual SNPs and BMD would not have reached statistical significance, but the association between the Ala348Thr in women with lumbar spine BMD would still be significant.

No information was available on causes of the fractures of our study participants, which prevented us from distinguishing between traumatic and non-traumatic, i.e. possible osteoporotic, fractures. However, since traumatic fractures are probably unrelated to BMD while non-traumatic fractures are likely associated with lower BMD values, a wider range in BMD values was realized in our study population by not excluding patients with traumatic fractures. Moreover, the lack of information on the cause of fractures was not essential for investigating the association between P2X7R SNPs and BMD, i.e. the risk of osteoporosis.

Recently performed genome-wide association studies (GWAS) have confirmed many previously identified specific genes associated with osteoporosis risk [37–39]. Although P2 receptor genes have been shown to be candidate genes for the development of osteoporosis, these genes were not identified by GWAS at a genome-wide significance level. Moreover, the effect sizes of SNPs are relatively small in a polygenetic trait such as BMD. However, current GWAS studies are best powered for SNPs with a population frequency in the range of 10 to 90 %. Therefore, a relatively rare polymorphisms such as most of the non-synonymous SNPs in the P2XR7 would likely have been missed in GWAS studies.

In conclusion, our results show that genetic aberration of P2X7R function is associated with BMD and osteoporosis risk in a cohort of fracture patients. Mapping P2X7R function genetically might therefore be a useful diagnostic tool for the management of osteoporosis in an early stage. Our findings warrant further observational studies in which fracture incidence as a major endpoint in relation to genetic variation in P2X7R function is prospectively monitored in addition to BMD.

## Electronic supplementary material

Below is the link to the electronic supplementary material.

ESM 1 (DOC 34.5 kb)

## References

[1] Lindsay R, Silverman SL, Cooper C, Hanley DA, Barton I, Broy SB, Licata A, Benhamou L, Geusens P, Flowers K, Stracke H, Seeman E (2001). Risk of new vertebral fracture in the year following a fracture. JAMA.

[2] Ross PD, Genant HK, Davis JW, Miller PD, Wasnich RD (1993). Predicting vertebral fracture incidence from prevalent fractures and bone density among non-black, osteoporotic women. Osteoporos Int.

[3] Gartland A, Hipskind RA, Gallagher JA, Bowler WB (2001). Expression of a P2X_7_ receptor by a subpopulation of human osteoblasts. J Bone Miner Res.

[4] Nakamura E, Uezono Y, Narusawa K, Shibuya I, Oishi Y, Tanaka M, Yanagihara N, Nakamura T, Izumi F (2000). ATP activates DNA synthesis by acting on P2X receptors in human osteoblast-like MG-63 cells. Am J Physiol Cell Physiol.

[5] Henriksen Z, Nissen N, Jorgensen NR (2006) Functional P2X7 purinergic receptors are expressed in differentiated human osteoblasts. J Bone Min res abstract SU208

[6] Buckley KA, Hipskind RA, Gartland A, Bowler WB, Gallagher JA (2002). Adenosine triphosphate stimulates human osteoclast activity via upregulation of osteoblast-expressed receptor activator of nuclear factor-kappa B ligand. Bone.

[7] Orriss IR, Knight GE, Ranasinghe S, Burnstock G, Arnett TR (2006). Osteoblast responses to nucleotides increase during differentiation. Bone.

[8] Jorgensen NR, Henriksen Z, Sorensen OH, Eriksen EF, Civitelli R, Steinberg TH (2002). Intercellular calcium signaling occurs between human osteoblasts and osteoclasts and requires activation of osteoclast P2X_7_ receptors. J Biol Chem.

[9] Li J, Liu D, Zhu Ke H, Duncan RL, Turner CH (2005). The P2X_7_ nucleotide receptor mediates skeletal mechanotransduction. J Biol Chem.

[10] Grol MW, Panupinthu N, Korcok J, Sims SM, Dixon SJ (2009). Expression, signaling, and function of P2X7 receptors in bone. Purinergic Signal.

[11] Orriss IR, Burnstock G, Arnett TR (2010). Purinergic signalling and bone remodelling. Curr Opin Pharmacol.

[12] Gartland AGA, Gallagher JA, Bowler WB (1999). Activation of P2X_7_ receptors expressed by human osteoclastoma modulates bone resorption. Calcif Tissue Int.

[13] Panupinthu N, Rogers JT, Zhao L, Pastor Solano-Flores L, Possmayer F, Sims SM, Dixon JS (2008). P2X7 receptors on osteoblasts couple to production of lysophosphatidic acid: a signaling axis promoting osteogenesis. J Cell Biol.

[14] Ke HZ, Qi H, Weidema AF, Zhang Q, Panupinthu N, Crawford DT, Grasser WA, Paralkar VM, Li M, Audoly LP, Gabel CA, Jee WS, Dixon SJ, Sims SM, Thompson DD (2003). Deletion of the P2X7 nucleotide receptor reveals its regulatory roles in bone formation and resorption. Mol Endocrinol.

[15] Li J, Liu D, Ke HZ, Duncan RL, Turner CH (2005). The P2X7 nucleotide receptor mediates skeletal mechanotransduction. J Biol Chem.

[16] Ohlendorff SD, Tofteng CL, Jensen J-EB, Petersen S, Civitelli R, Fenger M, Abrahamsen B, Hermann AP, Eiken P, Jorgensen NR (2007). Single nucleotide polymorphisms in the P2X_7_ gene are associated to fracture risk and to effect estrogen treatment. Pharmacogenet Genomics.

[17] Lise B, Husted TH, Liselotte Stenkjaer, Mette Carstens, Niklas R. Jorgensen, Bente L. Langdahl (2012) Functional polymorphisms in the p2x7 receptor gene are associated with osteoporosis. Bone. doi:10.1007/s00198-012-2035-5

[18] Mrazek F, Gallo J, Stahelova A, Petrek M (2009). Functional variants of the P2RX7 gene, aseptic osteolysis, and revision of the total hip arthroplasty: a preliminary study. Hum Immunol.

[19] Jorgensen NR, Husted LB, Skarratt KK, Stokes L, Tofteng CL, Kvist T, Jensen JE, Eiken P, Brixen K, Fuller S, Clifton-Bligh R, Gartland A, Schwarz P, Langdahl BL, Wiley JS Single-nucleotide polymorphisms in the P2X7 receptor gene are associated with post-menopausal bone loss and vertebral fractures. Eur J Hum Genet. doi:10.1038/ejhg.2011.253 10.1038/ejhg.2011.253 PMC3355253 22274585

[20] Gartland A, Skarratt KK, Hocking LJ, Parsons C, Stokes L, Jorgensen NR, Fraser WD, Reid DM, Gallagher JA, Wiley JS Polymorphisms in the P2X7 receptor gene are associated with low lumbar spine bone mineral density and accelerated bone loss in post-menopausal women. Eur J Hum Genet. doi:10.1038/ejhg.2011.245 10.1038/ejhg.2011.245 PMC3330223 22234152

[21] van Helden S, Cauberg E, Geusens P, Winkes B, van der Weijden T, Brink P (2007). The fracture and osteoporosis outpatient clinic: an effective strategy for improving implementation of an osteoporosis guideline. J Eval Clin Pract.

[22] Hansen T, Jakobsen KD, Fenger M, Nielsen J, Krane K, Fink-Jensen A, Lublin H, Ullum H, Timm S, Wang AG, Jorgensen NR, Werge T (2008). Variation in the purinergic P2RX(7) receptor gene and schizophrenia. Schizophr Res.

[23] Cabrini G, Falzoni S, Forchap SL, Pellegatti P, Balboni A, Agostini P, Cuneo A, Castoldi G, Baricordi OR, Di Virgilio F (2005). A His-155 to Tyr polymorphism confers to gain-of-function to the human P2X_7_ receptor of human leukemic lymphocytes. J Immunol.

[24] Stokes L, Fuller SJ, Sluyter R, Skarratt KK, Gu BJ, Wiley JS (2010). Two haplotypes of the P2X(7) receptor containing the Ala-348 to Thr polymorphism exhibit a gain-of-function effect and enhanced interleukin-1beta secretion. FASEB J.

[25] Roger S, Mei ZZ, Baldwin JM, Dong L, Bradley H, Baldwin SA, Surprenant A, Jiang LH (2009). Single nucleotide polymorphisms that were identified in affective mood disorders affect ATP-activated P2X7 receptor functions. J Psychiatr Res.

[26] Sun C, Chu J, Singh S, Salter RD (2009). Identification and characterization of a novel variant of the human P2X(7) receptor resulting in gain of function. Purinergic Signal.

[27] Gu BJ, Sluyter R, Skarratt KK, Shemon AN, Dao-Ung L-P, Fuller SJ, Barden JA, Clarke AL, Petrou S, Wiley JS (2004). An Arg^307^ to Gln polymorphism within the ATP-binding site causes loss of function of the human P2X_7_ receptor. J Biol Chem.

[28] Fernando SL, Saunders BM, Sluyter R, Skarratt KK, Wiley JS, Britton WJ (2005). Gene dosage determines the negative effects of polymorphic alleles of the P2X7 receptor on adenosine triphosphate-mediated killing of mycobacteria by human macrophages. J Infect Dis.

[29] Denlinger LC, Coursin DB, Schell K, Angelini G, Green DN, Guadarrama AG, Halsey J, Prabhu U, Hogan KJ, Bertics PJ (2006). Human P2X7 pore function predicts allele linkage disequilibrium. Clin Chem.

[30] Wiley JS, Dao-Ung L-P, Li C, Shemon AN, Gu BJ, Smart ML, Fuller SJ, Barden JA, Petrou S, Sluyter R (2003). An Ile-568 to Asn polymorphism prevents normal trafficking and function of the human P2X_7_ receptor. J Biol Chem.

[31] Roger S, Mei ZZ, Baldwin JM, Dong L, Bradley H, Baldwin SA, Surprenant A, Jiang LH Single nucleotide polymorphisms that were identified in affective mood disorders affect ATP-activated P2X7 receptor functions. J Psychiatr Res 44 (6): 347–355. doi:10.1016/j.jpsychires.2009.10.005 10.1016/j.jpsychires.2009.10.005 19931869

[32] Denlinger LC, Angelini G, Schell K, Green DN, Guadarrama AG, Prabhu U, Coursin DB, Bertics PJ, Hogan K (2005). Detection of human P2X_7_ nucleotide receptor polymorphisms by a novel monocyte pore assay predictive of alterations in lipopolysaccharide-induced cytokine production. J Immunol.

[33] Gu BJ, Zhang W, Worthington RA, Sluyter R, Dao-Ung P, Petrou S, Barden JA, Wiley JS (2001). A Glu-496 to Ala polymorphism leads to loss of function of the human P2X_7_ receptor. J Biol Chem.

[34] Wiley JS, Dao-Ung LP, Gu BJ, Sluyter R, Shemon AN, Li C, Taper J, Gallo J, Manoharan A (2002). A loss-of-function polymorphic mutation in the cytolytic P2X7 receptor gene and chronic lymphocytic leukaemia: a molecular study. Lancet.

[35] Genetos DC, Kephart CJ, Zhang Y, Yellowley CE, Donahue HJ (2007). Oscillating fluid flow activation of gap junction hemichannels induces ATP release from MLO-Y4 osteocytes. J Cell Physiol.

[36] Moran (2003). Arguments for rejecting the sequential Bonferroni in ecological studies. Oikos.

[37] Richards JB, Kavvoura FK, Rivadeneira F, Styrkarsdottir U, Estrada K, Halldorsson BV, Hsu YH, Zillikens MC, Wilson SG, Mullin BH, Amin N, Aulchenko YS, Cupples LA, Deloukas P, Demissie S, Hofman A, Kong A, Karasik D, van Meurs JB, Oostra BA, Pols HA, Sigurdsson G, Thorsteinsdottir U, Soranzo N, Williams FM, Zhou Y, Ralston SH, Thorleifsson G, van Duijn CM, Kiel DP, Stefansson K, Uitterlinden AG, Ioannidis JP, Spector TD (2009). Collaborative meta-analysis: associations of 150 candidate genes with osteoporosis and osteoporotic fracture. Ann Intern Med.

[38] Rivadeneira F, Styrkarsdottir U, Estrada K, Halldorsson BV, Hsu YH, Richards JB, Zillikens MC, Kavvoura FK, Amin N, Aulchenko YS, Cupples LA, Deloukas P, Demissie S, Grundberg E, Hofman A, Kong A, Karasik D, van Meurs JB, Oostra B, Pastinen T, Pols HA, Sigurdsson G, Soranzo N, Thorleifsson G, Thorsteinsdottir U, Williams FM, Wilson SG, Zhou Y, Ralston SH, van Duijn CM, Spector T, Kiel DP, Stefansson K, Ioannidis JP, Uitterlinden AG (2009). Twenty bone-mineral-density loci identified by large-scale meta-analysis of genome-wide association studies. Nat Genet.

[39] Estrada K, Styrkarsdottir U, Evangelou E, Hsu YH, Duncan EL, Ntzani EE, Oei L, Albagha OM, Amin N, Kemp JP, Koller DL, Li G, Liu CT, Minster RL, Moayyeri A, Vandenput L, Willner D, Xiao SM, Yerges-Armstrong LM, Zheng HF, Alonso N, Eriksson J, Kammerer CM, Kaptoge SK, Leo PJ, Thorleifsson G, Wilson SG, Wilson JF, Aalto V, Alen M, Aragaki AK, Aspelund T, Center JR, Dailiana Z, Duggan DJ, Garcia M, Garcia-Giralt N, Giroux S, Hallmans G, Hocking LJ, Husted LB, Jameson KA, Khusainova R, Kim GS, Kooperberg C, Koromila T, Kruk M, Laaksonen M, Lacroix AZ, Lee SH, Leung PC, Lewis JR, Masi L, Mencej-Bedrac S, Nguyen TV, Nogues X, Patel MS, Prezelj J, Rose LM, Scollen S, Siggeirsdottir K, Smith AV, Svensson O, Trompet S, Trummer O, van Schoor NM, Woo J, Zhu K, Balcells S, Brandi ML, Buckley BM, Cheng S, Christiansen C, Cooper C, Dedoussis G, Ford I, Frost M, Goltzman D, Gonzalez-Macias J, Kahonen M, Karlsson M, Khusnutdinova E, Koh JM, Kollia P, Langdahl BL, Leslie WD, Lips P, Ljunggren O, Lorenc RS, Marc J, Mellstrom D, Obermayer-Pietsch B, Olmos JM, Pettersson-Kymmer U, Reid DM, Riancho JA, Ridker PM, Rousseau F, Lagboom PE, Tang NL, Urreizti R, Van Hul W, Viikari J, Zarrabeitia MT, Aulchenko YS, Castano-Betancourt M, Grundberg E, Herrera L, Ingvarsson T, Johannsdottir H, Kwan T, Li R, Luben R, Medina-Gomez C, Th Palsson S, Reppe S, Rotter JI, Sigurdsson G, van Meurs JB, Verlaan D, Williams FM, Wood AR, Zhou Y, Gautvik KM, Pastinen T, Raychaudhuri S, Cauley JA, Chasman DI, Clark GR, Cummings SR, Danoy P, Dennison EM, Eastell R, Eisman JA, Gudnason V, Hofman A, Jackson RD, Jones G, Jukema JW, Khaw KT, Lehtimaki T, Liu Y, Lorentzon M, McCloskey E, Mitchell BD, Nandakumar K, Nicholson GC, Oostra BA, Peacock M, Pols HA, Prince RL, Raitakari O, Reid IR, Robbins J, Sambrook PN, Sham PC, Shuldiner AR, Tylavsky FA, van Duijn CM, Wareham NJ, Cupples LA, Econs MJ, Evans DM, Harris TB, Kung AW, Psaty BM, Reeve J, Spector TD, Streeten EA, Zillikens MC, Thorsteinsdottir U, Ohlsson C, Karasik D, Richards JB, Brown MA, Stefansson K, Uitterlinden AG, Ralston SH, Ioannidis JP, Kiel DP, Rivadeneira F Genome-wide meta-analysis identifies 56 bone mineral density loci and reveals 14 loci associated with risk of fracture. Nat Genet 44 (5):491–501. doi:10.1038/ng.2249 10.1038/ng.2249 PMC3338864 22504420

