# Supplementary material for: Association of P2X7 receptor polymorphisms with bone mineral density and osteoporosis risk in a cohort of Dutch fracture patients
Source: Osteoporos Int. 2012 Jul 10;24(4):1235–46. doi: 10.1007/s00198-012-2059-x (PMC3604588; doi:10.1007/s00198-012-2059-x)
Supplement: Supplementary file 1 — (DOC 34.5 kb) [file 198_2012_2059_MOESM1_ESM.doc]

Supplemental table 1: frequency of other fractures in study population

| Type of fracture | All subjects (% (N)) | Osteoporosis (% (N)) | Osteopenia (% (N)) | Normal BMD (% (N)) |
| --- | --- | --- | --- | --- |
| **Collar** | 3.8 (16) | 4.6 (5) | 2.8 (5) | 4.5 (6) |
| **Ribs** | 3.8 (16) | 0.9 (1) | 4.5 (8) | 5.3 (7) |
| **Lower leg** | 13.8 (58) | 17.6 (19) | 10.1 (18) | 15.8 (21) |
| **Foot** | 17.2 (72) | 13.0 (14) | 20.8 (37) | 15.8 (21) |
| **Ankle** | 22.4 (94) | 22.2 (24) | 20.2 (36) | 25.6 (34) |
| **Hand** | 18.1 (76) | 18.5 (20) | 16.3 (29) | 20.3 (27) |
| **Lower arm** | 13.8 (58) | 11.1 (12) | 18.5 (33) | 9.8 (13) |
| **Face** | 0.9 (4) | 0.9 (1) | 0.6 (1) | 1.5 (2) |
| **Knee** | 1.9 (8) | 3.7 (4) | 2.2 (4) | 0 (0) |
| **Shoulder** | 0.2 (1) | 0.9 (1) | 0 (0) | 0 (0) |
| **Pubic** | 2.1 (9) | 3.7 (4) | 2.2 (4) | 0.7 (1) |
| **Pelvic** | 1.4 (6) | 1.8 (2) | 1.7 (3) | 0.7 (1) |
| **Skull** | 0.2 (1) | 0.9 (1) | 0 (0) | 0 (0) |
